# Screening of Anti-Lipase Components of *Artemisia argyi* Leaves Based on Spectrum-Effect Relationships and HPLC-MS/MS

**DOI:** 10.3389/fphar.2021.675396

**Published:** 2021-05-07

**Authors:** Yaqing Chang, Dan Zhang, Guiya Yang, Yuguang Zheng, Long Guo

**Affiliations:** ^1^ Traditional Chinese Medicine Processing Technology Innovation Center of Hebei Province, Hebei University of Chinese Medicine, Shijiazhuang, China; ^2^ Hebei Chemical and Pharmaceutical College, Shijiazhuang, China

**Keywords:** *Artemisia argyi* leaves, anti-lipase activity, spectrum-effect relationships, pancreatic lipase, HPLC-MS/MS

## Abstract

Pancreatic lipase is a key lipase for triacylglyceride digestion and absorption, which is recognized as a promising target for treatment of metabolic disorders. Natural phytochemicals are hopeful sources for pancreatic lipase inhibitors. The leaves of *Artemisia argyi* H.Lév. and Vaniot (AL) is commonly used as herbal medicine or food supplement in China and other Asian countries for hundreds of years. AL mainly contains essential oils, phenolic acids, flavonoids and terpenoids, which exhibit many pharmacological activities such as antioxidant, anti-inflammatory, antimicrobial, analgetic, anti-cancer, anti-diabetes and immunomodulatory effects. However, the anti-lipase activity of AL was lack of study and the investigation of anti-lipase ingredients from AL was also insufficient. In the present study, the anti-lipase activity of AL was evaluated *in vitro* and the potentially pancreatic lipase inhibitors of AL were investigated. High performance liquid chromatography was used to establish fingerprints of AL samples, and fifteen peaks were selected. The anti-lipase activities of AL samples were evaluated by a pancreatic lipase inhibition assay. Then, the spectrum-effect relationships between fingerprints and pancreatic lipase inhibitory activities were investigated to identify the anti-lipase constitutes in AL. As the results, four caffeoylquinic acids, which were identified as neochlorogenic acid, chlorogenic acid, isochlorogenic acid B, and isochlorogenic acid A by high performance liquid chromatography coupled with quadrupole time-of-flight mass spectrometry, were selected as potential pancreatic lipase inhibitors in AL. Moreover, anti-lipase activity assessment and molecular docking study of the four compounds were performed to validate the potential lipase inhibitors in AL. The results revealed that the four caffeoylquinic acids in AL as bioactive compounds displayed with anti-lipase activity. The present research provided evidences for the anti-lipase activity of AL, and suggested that some bioactive compounds in AL could be used as lead compounds for discovering of new pancreatic lipase inhibitors.

## Introduction

Hyperlipidemia is a chronic, progressive and systemic disease characterized by the lipid metabolism disorders, which is a significant modifiable risk factor for cardiovascular and metabolic diseases (Ray et al., 2019). Drug intervention of lipid metabolism provides a credible method for prevention or treatment of metabolic disorders (Zeng et al., 2018). Pancreatic lipase is a key enzyme responsible for hydrolyzing triacylglycerides in the duodenum, which has been discovered as the crucial target that regulates lipid absorption (Hou et al., 2020b). The pancreatic lipase is secreted from the pancreas, and it has been demonstrated that inhibition on pancreatic lipase and regulation of lipid absorption is an effective approach for discovering new agents for treatment of metabolic disorders (Birari and Bhutani, 2007). Orlistat, which is a hydrogenated derivative of lipstatin, is the only approved pancreatic lipase inhibitor so far. Orlistat exhibits potent anti-lipase effect, but it can cause various side effects, such as oily spotting, fecal incontinence, flatus with discharge and abdominal cramping (Navarro Del Hierro et al., 2021). Thus, discovery and development of safe and effective pancreatic lipase inhibitors are urgently needed.

Recently, there has been a great interest in the screening of potential pancreatic lipase inhibitors from herbal medicines, because that herbal medicines have shown satisfying safety profiles in long-term medical treatments (Sham et al., 2014). Up to now, several herbal medicines, such as Citri Reticulatae Pericarpium and Mori Radicis Cortex, have been demonstrated with strong inhibition on pancreatic lipase for the regulation of lipid metabolism, which are applied to treat hyperlipidemia and other metabolic diseases in clinic (Hou et al., 2018; Zeng et al., 2018). The leaves of *Artemisia argyi* H.Lév. and Vaniot (AL), a common herbal medicine for treatment of hemorrhage, dysmenorrhea, abdominal pain eczema and skinitch (Guo et al., 2019), which is mainly distributed in China, Korea, Mongolia, and Japan (Abad et al., 2012). AL is also used as a food ingredient due to its delicious flavor and characteristic smell (Xiao et al., 2019)., AL is consumed as a condiment and colorant for the traditional food “Qingtuan” in China, and AL is also used as an additive in dietary food to enhance the flavor and nutrition in Japan (Abad et al., 2012). Previous investigations have been reported that AL contains many bioactive compounds such as essential oil, phenols, flavonoids and terpenoids, which possess multiple bioactivities, such as antioxidant, anti-inflammatory, antibacterial, antiviral, analgetic, anti-hypertensive, hypoglycemic, hemostatic and immunoregulatory effects (Huang et al., 2012; Dib and El Alaoui-Faris, 2019; Zhang and Lv, 2019). However, the anti-lipase activity of AL was lack of study and the investigation of anti-lipase compounds from AL was also insufficient.

It is inefficient to discover bioactive compounds from herbal medicines based on the commonly methods of extraction, purification, structure identification and bioassay. (Jiang et al., 2010; Quan et al., 2019; Gong et al., 2020). To overcome this shortcoming, a new and reliable method named spectrum-effect relationships analysis was employed to investigate the correlations between pharmacodynamics and chemical components of herbal medicines (Yang et al., 2016; Liu et al., 2019). Chromatographic fingerprints could characterize the chemical components of herbal medicines, which is an effective method for uniformity and quality evaluation of herbal medicines. Combining with the results of pharmacodynamics research, the chromatographic fingerprint data could be applied to analysis the spectrum-effect relationships to screen out the bioactive compounds from herbal medicines (Jiang et al., 2018; Zhang et al., 2019). In recent years, the spectrum-effect relationships analysis has been used to evaluate the quality and discover bioactive components of herbal medicines as an efficient strategy (Morlock et al., 2014; Xiao et al., 2018).

In this present study, the chromatographic fingerprints of AL samples were established using high performance liquid chromatography (HPLC), and the anti-lipase activities of AL samples were evaluated by the pancreatic lipase inhibition assay. Combined with the data of fingerprints and anti-lipase activities, the spectrum-effect relationships were performed to screen anti-lipase constitutes in AL by Pearson correlation analysis and partial least squares regression (PLSR). The selected pancreatic lipase inhibitors were further identified by high performance liquid chromatography coupled with quadrupole time-of-flight mass spectrometry (HPLC-Q/TOF-MS). In addition, the anti-lipase activity of the selected compounds was validated and *in silico* molecular docking research was performed to optimize and predict the pancreatic lipase inhibitors in AL.

## Materials and Methods

### Materials and Reagents

Twenty-two batches of AL were collected from different areas in China, and the sample origins are provided in Supplementary Table S1. The voucher specimens, identified by Associate Professor Long Guo have been deposited in Traditional Chinese Medicine Processing Technology Innovation Center of Hebei Province, Hebei University of Chinese Medicine.

Reference standards of neochlorogenic acid, chlorogenic acid, isochlorogenic acid B and isochlorogenic acid A were purchased from Chengdu Must Bio-Technoligy Co., Ltd. (Chengdu, China). The purities of these compounds were determined to be higher than 98% by high performance liquid chromatography with diode array detector. The chemical structures of the four compounds are shown in Supplementary Figure S1. Porcine pancreatic lipase (type II) and 4-methylumbelliferyl oleate were purchased from Sigma-Aldrich (St Louis, MO, United States).

HPLC grade methanol, acetonitrile and formic acid were obtained from Fisher Scientific (Pittsburgh, PA, United States). Ultrapure water was prepared by a Synergy water purification system (Millipore, Billerica, United States). Other chemicals and reagents were of analytical grade.

### HPLC-DAD and HPLC-Q/TOF-MS Conditions

The HPLC-DAD analysis was performed on a Shimadzu LC-2030 system comprised an auto-sampler, a binary pump, a thermostatically controlled column apartment and a photo-diode array detector (Shimadzu Seisakusho, Kyoto, Japan). Chromatographic separation was conducted on an Agilent ZORBAX SB C18 column (4.6 × 50 mm, 1.8 μm). The mobile phase consists of 0.1% formic-water (A) and acetonitrile (B) with a gradient elution as follows: 0–5 min, 10% B; 5–10 min, 10–15% B; 10–22 min, 15–22% B; 22–27 min, 22–25% B; 27–37 min, 25–30% B; 37–44 min, 30% B; 44–47 min, 30–42% B; 47–52 min, 42–53%. The flow rate was maintained at 0.4 ml/min, and the column temperature was set at 25°C. The detection wavelength was set at 340 nm.

The HPLC-Q/TOF-MS analysis was performed on an Agilent 1290 UHPLC system coupled with an Agilent 6545 quadrupole time-of-flight mass spectrometer system (Agilent Technologies, Santa Clara, CA, United States). Chromatographic separation was also performed on an Agilent ZORBAX SB C18 column (4.6 × 50 mm, 1.8 μm) and the HPLC chromatographic conditions were the same as HPLC-DAD analysis. The MS acquisition parameters were as follows: drying gas (N_2_) temperature, 320°C; sheath gas temperature, 350°C; drying gas (N_2_) flow rate, 10.0 L/min; sheath gas flow (N_2_) rate, 11 L/min; nebulizer gas pressure, 35 psi; capillary voltage, 3500 V; fragmentor voltage, 135 V; collision energy, 40 eV. The analysis was operated in negative mode with the mass range of m/z 120–1,000 Da. Data acquisition was conducted on MassHunter Workstation (Agilent Technologies, United States).

### Sample Preparation

AL samples were powdered and screened through 40 mesh sieves. Each sample powder (0.2 g) was accurately weighed and thoroughly mixed with 75% (v/v) methanol (10 ml), then extracted by ultrasonator for 30 min. The extracted solution was centrifuged at 13,000 r/min for 10 min. For HPLC-DAD and HPLC-Q/TOF-MS analysis, the supernatant was injected into the HPLC instrument. For pancreatic lipase inhibition assay, 600 μl of the supernatant was evaporated to dryness and dissolved in 600 μl Tris-HCl buffer to remove the methanol, since the methanol might inactivated the pancreatic lipase.

### HPLC Fingerprints

#### Method Validation

To ensure the reliability of the HPLC-DAD method used for AL samples analysis, the precision, repeatability and stability of the HPLC method was validated. The precision was determined by the intra- and inter-day variations. For intra-day precision, one AL sample was analyzed for six times within the same day, while for inter-day precision, the sample was examined in duplicates for consecutive three days. For the repeatability test, six replicates of the same AL sample was prepared and analyzed. To confirm the stability, the same AL sample was stored at room temperature and analyzed at 0, 2, 6, 8, 12 and 24 h. The relative standard deviations (RSDs) of peak areas for the fifteen common peaks were used to evaluate the precision, repeatability and stability of the established HPLC method.

#### Establishment and Evaluation of HPLC Fingerprints

Twenty-two batches of AL samples were analyzed to obtain the chromatograms containing the peak areas and relative retention times, and the chromatograms data were saved as CDF format. Using the Similarity Evaluation System for Chromatographic Fingerprint of Traditional Chinese Medicine (version 2004 A), the HPLC fingerprints of AL samples were matched automatically, and the reference fingerprint was formed with the median method by comparison of the chromatograms of twenty-two batches of AL samples. The similarities between the reference fingerprint and the chromatograms of different AL samples were calculated.

### Pancreatic Lipase Inhibition Assay

The inhibitory activities of AL samples against pancreatic lipase were investigated using 4-methylumbelliferyl oleate as a substrate, according to the previous method with little modification (Kurihara et al., 2003). The AL samples, pancreatic lipase and 4-methylumbelliferyl oleate were prepared in Tris-HCl buffer solution (13 mM Tris-HCl, 150 mM NaCl, 1.3 mM CaCl_2_, pH 8.0). 25 µl of AL sample solution (at different concentrations) and 25 µl of pancreatic lipase solution (1 mg/ml) were added into a black bottom 96-well plate. After pre-incubation at 37°C for 10 min, the reaction was started by addition of 50 µl of 4-methylumbelliferyl oleate solution (1 mM). After incubation at 37°C for 20 min, the reaction was stopped by adding 100 μl of 0.1 M citrate buffer solution (pH 4.2). The amount of 4-methylumbelliferone released by the pancreatic lipase was measured with a fluorometrical microplate reader at an excitation wavelength of 355 nm and an emission wavelength of 460 nm. The control sample was prepared by adding buffer solution instead of tested sample. The background sample was prepared by replacing tested sample with the same volume of buffer solution. The blank sample was prepared by adding buffer solution instead of pancreatic lipase solution. All experiments were repeated three times. The inhibition of pancreatic lipase activity was calculated as follows:

$$\text{Inhibition}\left(\text{\%}\right)=\left(1-\frac{\text{test sample}-\text{background sample}}{\text{control sample}-\text{blank sample}}\right)\times 100$$

The pancreatic lipase inhibitory activity of AL samples were evaluated by the IC_50_ values (the concentration of the sample that inhibited 50% the activity of the pancreatic lipase), and the IC_50_ were calculated by a logarithmic regression curve and expressed as mg/mL methanol extracts equivalents.

To confirm the spectrum-effect relationships analysis result, the pancreatic lipase inhibitory activities of the potential anti-lipase ingredients, including neochlorogenic acid, chlorogenic acid, isochlorogenic acid B and isochlorogenic acid A were determined by the above pancreatic lipase inhibition assay, and the IC_50_ value of the four components were also calculated.

### Spectrum-Effect Relationship Analysis

#### Pearson Correlation Analysis

Pearson correlation analysis is a multivariate statistical model, which is applied to extract factors that have the greatest impact on the outcome variables and maximize the relationships between the two sets of variables (Armstrong et al., 2005). Taking the Pearson correlation coefficient as an index, the fifteen common peaks areas in the HPLC fingerprints of AL samples were recognized as one set of variables, and the anti-lipase activities (IC_50_ values) as the other set. The correlations between common peaks and IC_50_ values were analyzed by SPSS 18.0 statistics software (SPSS Inc., Chicago, IL, United States).

#### Partial Least Squares Regression Analysis

PLSR analysis is a multivariate regression model combining multivariate data fusion and principal component analysis (Svante et al., 2001). In this study, PLSR was used to model the correlation between the common peaks and anti-lipase activities. The fifteen common peak areas were set as the independent X variables, and the anti-lipase activities (IC_50_ values) were set as dependent Y variables. After extraction of principal components, the linear relationships between anti-lipase activities and common peaks were displayed by the PLSR model. The regression coefficients were considered as the index to reveal the relative impact of the predictor variables on the response variable for PLSR model. The PLSR was performed by Simca-P 14.0 software (Umetrics, Umea, Sweden).

### Molecular Modeling and Docking Study

An *in silico* protein-ligand docking software AutoDock 4.2 program was performed to analyze binding affinities of ligands to pancreatic lipase and predict the possible binding sites based on the standard procedures (Eswaramoorthy et al., 2021). The structure of pancreatic lipase (PDB ID: 1LPA) was obtained from Protein Data Bank. Unnecessary substructures and water molecules in pancreatic lipase were removed, and hydrogen atoms were added. The gasteiger charges of each atom of pancreatic lipase were calculated. Run AutoGrid to get grid maps. The number of runs was set as 100 by Lamarckian genetic algorithm to give docked conformations.

## Results

### HPLC Fingerprints

#### Optimization of HPLC Condition

In order to achieve a rapid and efficient separation of AL samples, several HPLC conditions, including mobile phases (water-methanol, water-acetonitrile, formic acid water-methanol and formic acid water-acetonitrile), flow rates (0.3, 0.4, and 0.5 ml/min), and column temperatures (20°C, 25°C, and 30°C) were optimized. The results indicated that formic acid water-acetonitrile was the best mobile phase for separation of the analystes. The optimal column temperature was 25°C and optimal flow rate was 0.4 ml/min in this present study.

#### Method Validation

The precision, repeatability and stability of the established HPLC-DAD method were validated. As shown in Supplementary Table S2, the intra-day and inter-day precision (RSDs) of peak areas for the fifteen common peaks were less than 2.7 and 2.9%, respectively. The repeatability presented as RSDs was less 2.6%, and the stability was less than 2.8%. The method validation results demonstrated that the established HPLC-DAD method is suitable for analysis of AL samples.

#### HPLC Fingerprints and Similarity Analysis

**FIGURE 1:**
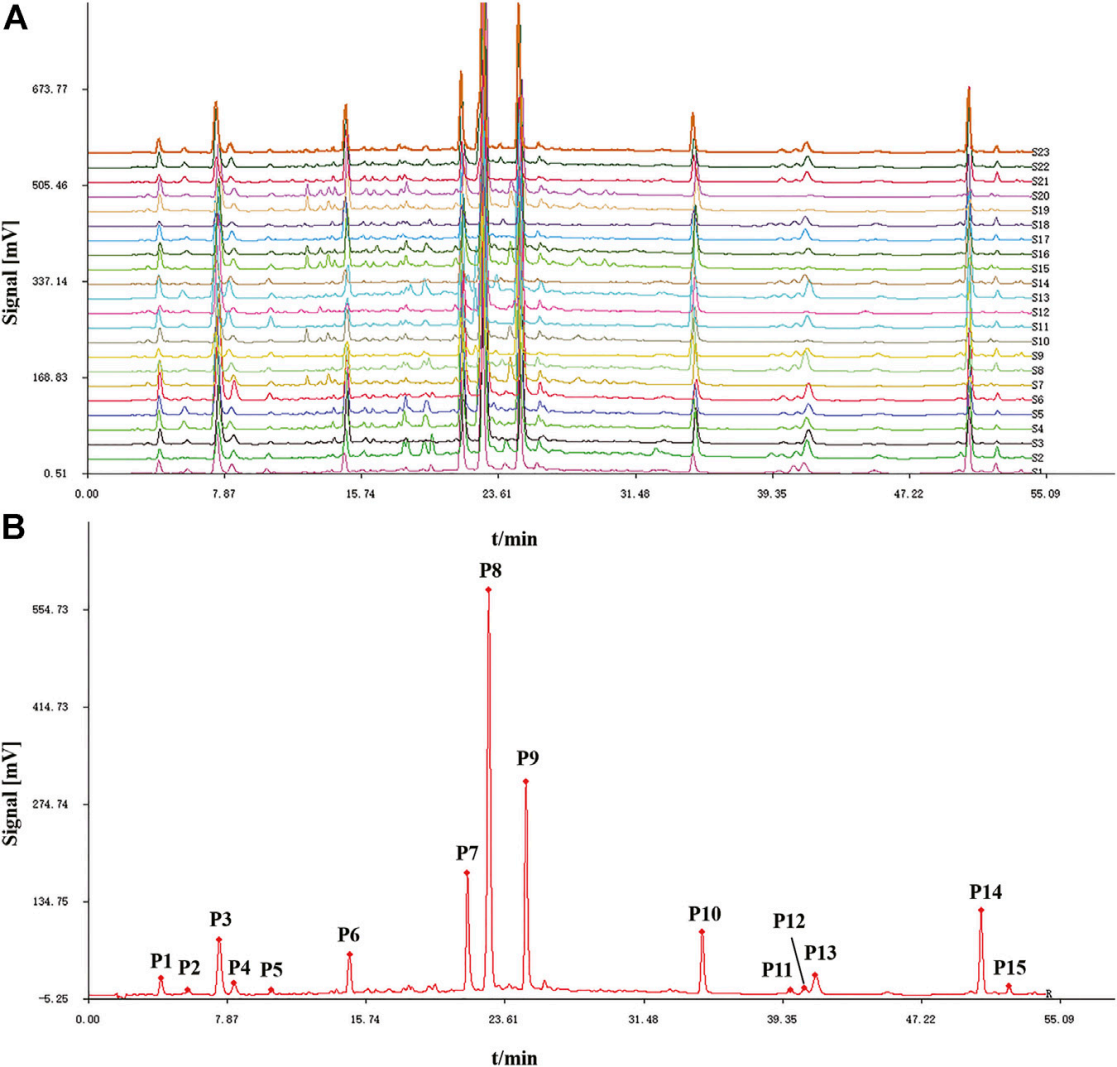
HPLC fingerprints **(A)** and reference fingerprint **(B)** of twenty-two batches (S1-S22) of *Artemisia argyi* leaves.

Twenty-two batches of AL samples collected from different areas were analyzed by HPLC-DAD using the optimized condition. Then, the HPLC fingerprints were established based on the chromatograms of AL samples by similarity evaluation software (Similarity Evaluation System for Chromatographic Fingerprint of Traditional Chinese Medicine). The chromatographic fingerprints of AL samples are shown in Figure 1A, and the reference fingerprint is displayed in Figure 1B. Fifteen peaks that existed in all the AL samples with good segregation and resolution were recognized as the common peaks, which indicated the similarity among various samples. The similarities between the HPLC chromatograms of AL samples and the reference fingerprint were compared, and the similarity values were calculated using the correlative coefficient and the cosine value of vectorial angle by the similarity evaluation software. As shown in Table 1, the similarity values between HPLC fingerprint of each AL sample and the reference fingerprint were in the range of 0.900–0.997. The similarity analysis results indicated that different batches of AL samples had similar chemical compositions, and the origin might not be the main factor that affects the quality diversity of AL samples.

**TABLE 1:** Similarities of twenty-two batches (S1-S22) of *Artemisia argyi* leaves collected from different areas.

NO.	Similarity	NO.	Similarity
S1	0.942	S12	0.931
S2	0.997	S13	0.900
S3	0.970	S14	0.984
S4	0.971	S15	0.985
S5	0.982	S16	0.994
S6	0.977	S17	0.900
S7	0.961	S18	0.979
S8	0.902	S19	0.978
S9	0.980	S20	0.922
S10	0.955	S21	0.968
S11	0.978	S22	0.978

### Anti-Lipase Activity

**FIGURE 2:**
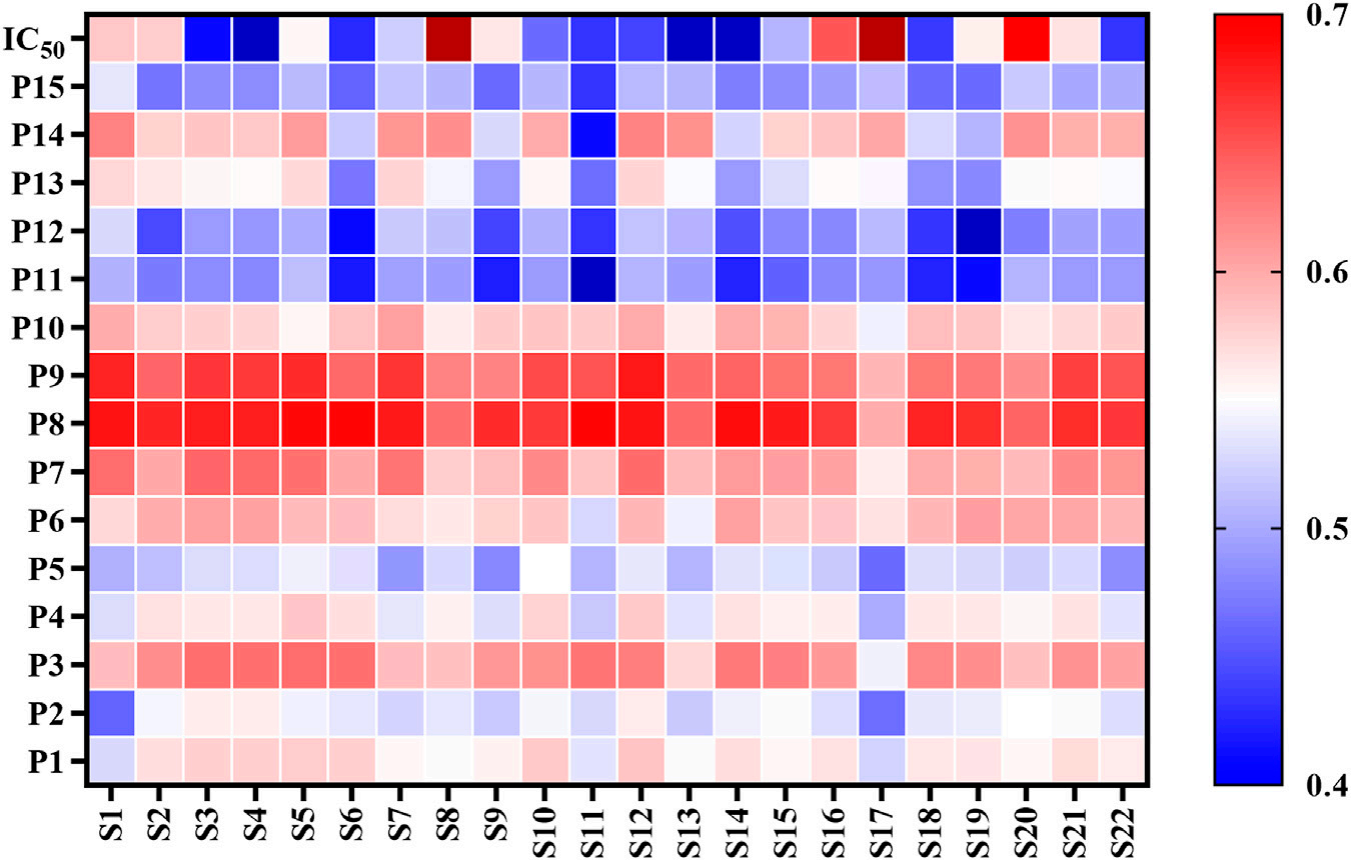
Heatmap analysis of fifteen common peaks (P1-P15) areas and anti-lipase activities (IC_50_ values of pancreatic lipase) of twenty-two batches (S1-S22) of *Artemisia argyi* leaves. Red represents higher contents and blue indicates lower contents.s

Most of the bioactive studies of AL are focused on the antioxidant and anti-inflammatory effects (Yun et al., 2016; Han et al., 2017; Xia et al., 2019). To the best of our knowledge, there are no reports about the anti-lipase activities of AL. In this work, the pancreatic lipase inhibitory capacities of twenty-two batches of AL samples collected from different areas were evaluated by pancreatic lipase inhibition assay. The results showed that AL samples inhibited pancreatic lipase in a concentration dependent manner with IC_50_ in the range from 1.92 to 10.29 mg/ml (Table 2). The heatmap (Figure 2) was adopted to provide the presentation of the common peak areas and anti-lipase activity difference of AL samples from different origins. It could be noted that the anti-lipase activities of AL samples showed significant differences, and the peak areas of bioactive ingredients were also different to some extent. The differences of anti-lipase activities might be due to the presence of various bioactive constituents in AL samples. Therefore, it is necessary to investigate the relationships between the bioactive compounds and anti-lipase activities of AL samples, and find the potential anti-lipase constituents through spectrum-effect relationships analysis.

**TABLE 2:** Inhibitory effects (IC_50_ values) of twenty-two batches (S1-S22) of *Artemisia argyi* leaves on pancreatic lipase.

NO.	IC_50_ (mg/ml)	No	IC_50_ (mg/ml)
S1	5.99 ± 0.08	S12	4.54 ± 0.12
S2	5.96 ± 0.07	S13	8.50 ± 0.13
S3	4.22 ± 0.06	S14	3.89 ± 0.12
S4	1.92 ± 0.05	S15	5.21 ± 0.10
S5	5.69 ± 0.04	S16	6.66 ± 0.18
S6	4.39 ± 0.02	S17	10.29 ± 0.21
S7	5.36 ± 0.00	S18	4.49 ± 0.07
S8	8.80 ± 0.05	S19	5.75 ± 0.16
S9	5.80 ± 0.11	S20	7.19 ± 0.18
S10	4.77 ± 0.06	S21	5.83 ± 0.01
S11	4.45 ± 0.14	S22	4.45 ± 0.02

### Spectrum-Effect Relationship Analysis

#### Pearson Correlation Analysis

**FIGURE 3:**
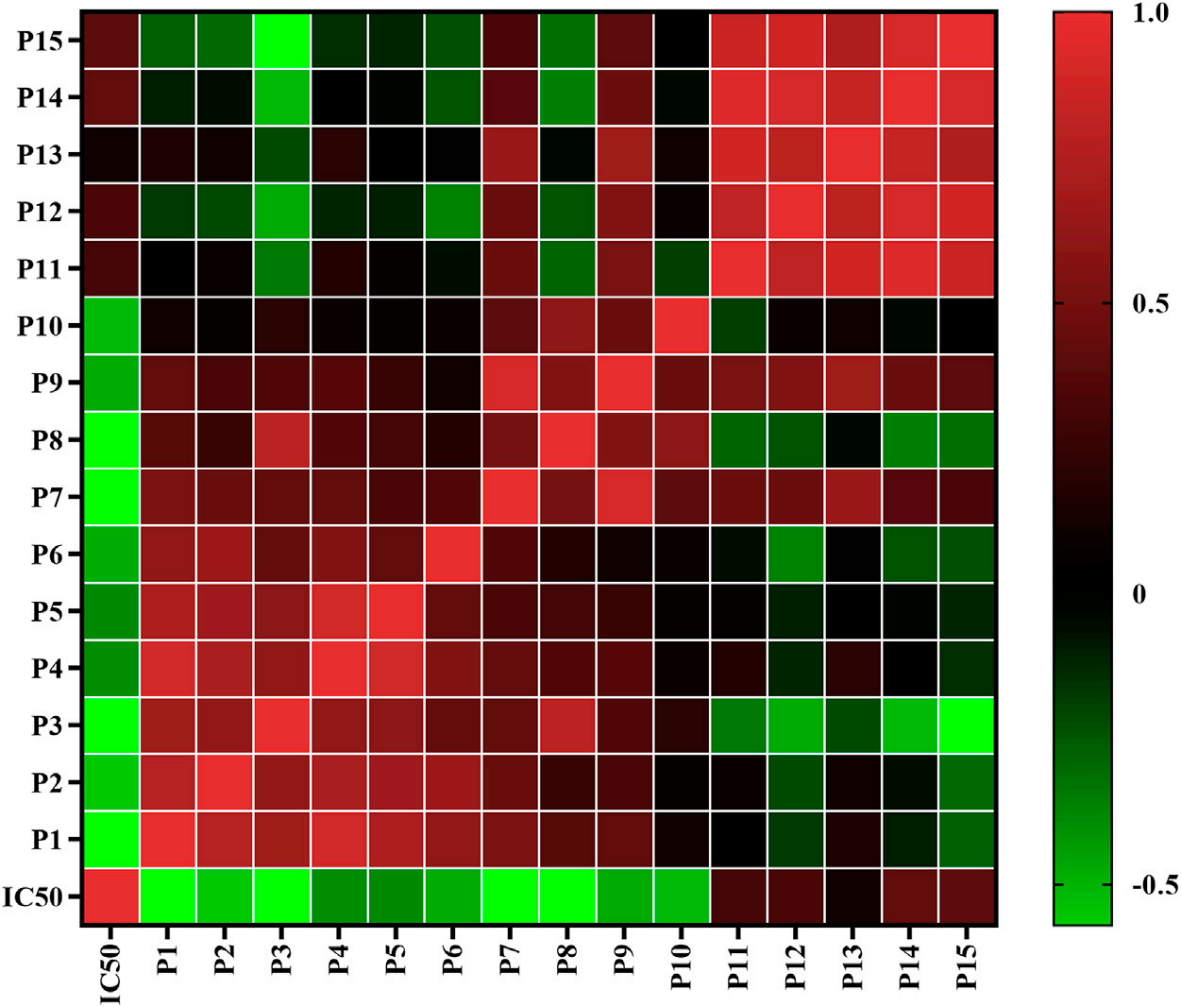
Heatmap analysis of Pearson correlation of fifteen common peaks (P1-P15) areas and anti-lipase activities (IC_50_ values of pancreatic lipase). Red represents positive correlated and green indicates negative correlated.

Pearson correlation analysis was firstly applied to study the spectrum-effect relationships between the IC_50_ values of pancreatic lipase inhibitory activities and the fifteen common peak areas of different AL samples. Pearson correlation coefficients of the fifteen common peaks are shown in Figure 3. It was observed that ten common peaks, P3, P8, P1, P7, P2, P10, P6, P9, P4 and P5 were negatively correlated to the IC_50_ values, which indicated these common peaks had strong inhibitory effects on pancreatic lipase. The correlation coefficients of P3, P8, P1, P7, P2, P10, P6, P9, P4 and P5 were −0.7920, −0.7690, −0.5820, −0.5710, −0.5610, −0.5240, −0.4860, −0.4730, −0.3960 and, −0.3900, respectively. The higher the absolute value of correlation coefficient was, the stronger anti-lipase effect the common peak had. While peaks P11, P12, P13, P14 and P15 showed positive correlation to the IC_50_ values, indicating that when the areas of these peaks increased the capability on anti-lipase activities would be weaker.

#### Partial Least Squares Regression Analysis

**FIGURE 4:**
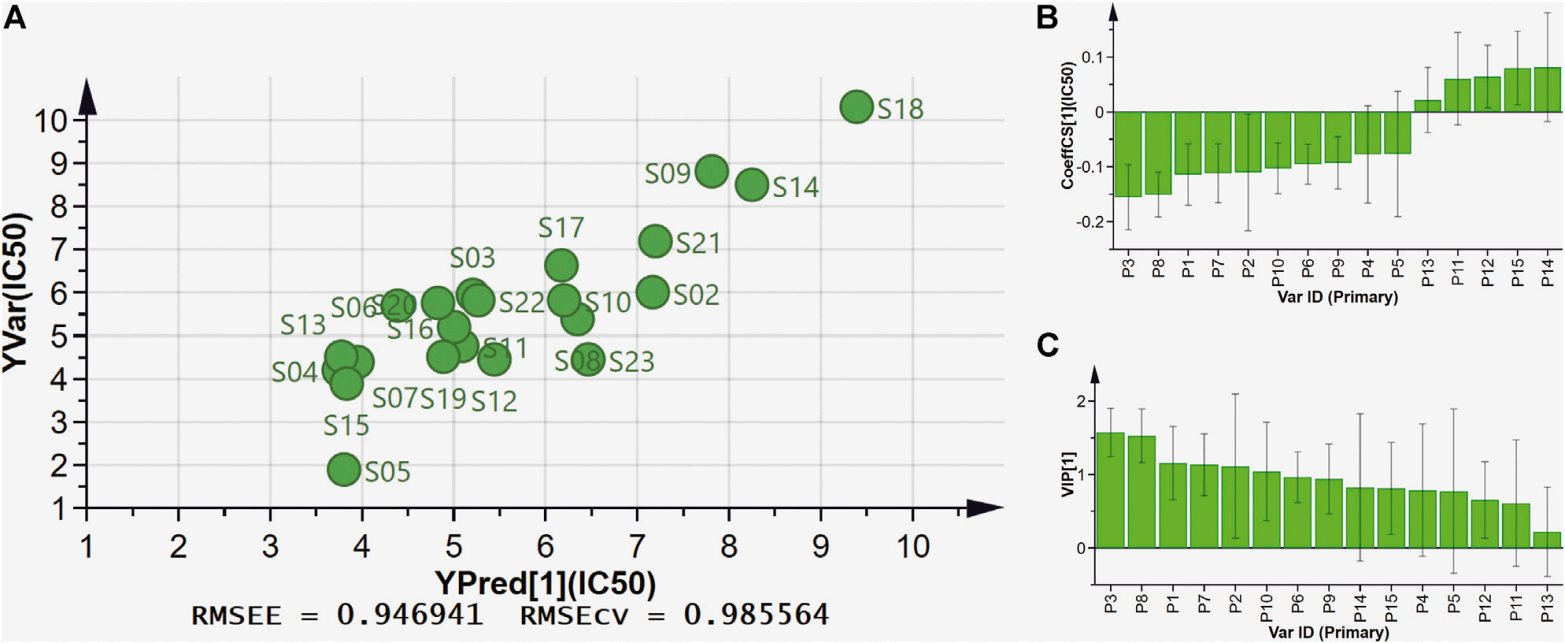
The results of spectrum-effect relationships by PLSR model. **(A)** PLSR linear regression; **(B)** Regression coefficients between fifteen common peaks and anti-lipase activities (IC_50_ values of pancreatic lipase); **(C)** VIP values of fifteen common peaks.

PLSR can fully use of data information, and is widely used in spectrum-effect relationships analysis. In this present work, PLSR was further performed to investigate the spectrum-effect relationships between anti-lipase activities and common peaks, and find pancreatic lipase inhibitors of AL samples. As shown in Figure 4A, the fifteen common peak areas were set as the independent X variables and the IC_50_ values of pancreatic lipase inhibitory activities of AL samples were taken as the dependent Y variables, and the PLSR models were established sequentially. The regression coefficients of the fifteen common peaks were calculated. As shown in Figure 4B, the ten common peaks P3, P8, P1, P7, P2, P10, P6, P9, P4, and P5 showed negative correlation to the IC_50_ values, which indicated that the anti-lipase activity increased with the increasing areas of these common peaks. The inhibitions of the ten common peaks on pancreatic lipase were as the following order: P3 > P8 > P1 > P7 > P2 > P10 > P6 > P9 > P4 > P5, which was consistent with the results obtained by Pearson correlation analysis. The Variable Importance for the Projection (VIP) values of the fifteen common peaks were also calculated. The VIP values represent the importance of the variables, and common peaks with VIP values greater than 1.0 could be considered to be responsible for anti-lipase activity. As shown in Figure 4C, the VIP values of P3, P8, P1, and P7 were 1.5742, 1.5279, 1.5778, and 1.1351 respectively, greater than 1.0. Combining the results of Pearson correlation analysis and PLSR, the four common peaks P3, P8, P1, and P7 were considered as potential pancreatic lipase inhibitors of AL.

### Identification of Fifteen Common Peaks in *Artemisia argyi* Leaves by HPLC-Q/TOF-MS

**FIGURE 5:**
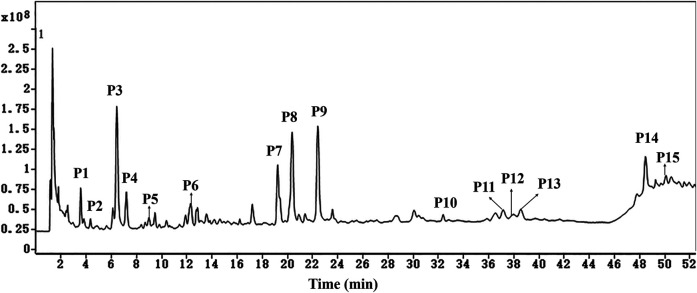
The typical total ion chromatogram of AL sample in negative ion mode. P1, neochlorogenic acid; P2, quinic acid; P3, chlorogenic acid; P4, cryptochlorogenic acid; P5, caffeic acid; P6, 5-feruoyl quinic acid; P7, isochlorogenic acid B; P8, isochlorogenic acid A; P9, isochlorogenic acid C; P10, 3,4,5-tri-*O*-caffeoylquinic acid; P11, hispidulin; P12, centaureidin; P13, jaceosidin; P14, eupatilin; P15, casticin.

The fifteen common peaks (P1-P15) in HPLC fingerprints of AL were further identified by HPLC-Q/TOF-MS, and the typical total ion chromatogram of AL sample in negative ion mode is illustrated in Figure 5. Fifteen compounds including two organic acids (P2 and P5), seven caffeoylquinic acids (P1, P3, P4, P7, P8, P9, and P10), one feruoylquinic acid (P6) and five methoxylated flavones (P11, P12, P13, P14, and P15) were identified or tentatively characterized. The HPLC-Q/TOF-MS information such as retention time, chemical formula, ppm errors and main fragment ions is summarized in Table 3.

**TABLE 3:** Identification of fifteen common peaks in *Artemisia argyi* leaves by HPLC-Q/TOF-MS.

Peak	Retention time (min)	[M−H]^−^ (*m/z*)	Formula	Error (ppm)	Fragment ions (*m/z*)	Identification
P1	3.55	353.0877	C_16_H_18_O_9_	−0.36	191.0560, 179.0347, 161.0241, 135.0452, 127.0400, 111.0450, 93.0344	Neochlorogenic acid
P2	6.44	191.0560	C_7_H_12_O_6_	−0.62	191.0562, 173.0451, 127.0402, 85.0297, 59.0142	Quinic acid
P3	6.54	353.0875	C_16_H_18_O_9_	−0.85	191.0560, 173.0453, 161.0245, 135.0449, 127.0400, 111.0450, 93.0345	Chlorogenic acid
P4	7.17	353.0875	C_16_H_18_O_9_	−0.89	353.0877, 173.0453, 135.0449, 111.0450, 85.0296, 59.0142	Cryptochlorogenic acid
P5	8.97	179.0348	C_9_H_8_O_4_	−0.91	179.035, 135.0450, 134.0369, 107.0503, 79.0551	Caffeic acid
P6	11.83	367.1032	C_17_H_20_O_9_	−0.70	367.1033, 191.0559, 173.0458, 149.0607, 134.0369	5-Feruoyl quinic acid
P7	19.23	515.1194	C_25_H_24_O_12_	0.15	515.1191, 353.0874, 335.0768, 299.0558, 191.0559, 173.0453, 161.0241, 135.0448	Isochlorogenic acid B
P8	20.50	515.1192	C_25_H_24_O_12_	0.05	515.1190, 353.0873, 335.0770, 191.0560, 179.0345, 161.0241, 135.0442	Isochlorogenic acid A
P9	22.56	515.1195	C_25_H_24_O_12_	−0.03	515.1194, 191.0560, 173.0453, 155.0345, 135.0450, 111.0450, 93.0344, 71.0140	Isochlorogenic acid C
P10	32.38	677.1507	C_34_H_30_O_15_	−0.96	677.1513, 515.1188, 353.0876, 191.0559, 173.0454, 135.0449	3,4,5-Tri-*O*-caffeoylquinic acid
P11	37.16	299.0560	C_16_H_12_O_6_	−0.39	299.0562, 284.0318, 255.0298, 227.0346, 183.0447, 136.9878, 94.0059	Hispidulin
P12	37.94	359.0771	C_18_H_16_O_8_	−0.56	344.0531, 329.0294, 314.0070, 301.0349, 286.0114, 258.0167, 242.0217, 214.0266	Centaureidin
P13	38.55	329.0666	C_17_H_14_O_7_	−0.37	329.0666, 299.0196, 271.0246, 243.0294, 227.0344, 199.0399, 171.0449, 133.0291, 65.0035	Jaceosidin
P14	48.47	343.0821	C_18_H_16_O_7_	−0.83	343.0823, 313.0351, 298.0115, 285.0401, 270.0165, 242.0216, 214.0267, 163.0035, 147.0447, 132.0215, 65.0035	Eupatilin
P15	50.11	373.0927	C_19_H_18_O_8_	−0.62	373.0929, 343.0453, 328.0223, 312.0987, 285.0037, 257.0089, 213.0190, 185.0237	Casticin

The above spectrum-effect relationships results illustrated that the common peaks P3, P8, P1, and P7 were potential pancreatic lipase inhibitory compounds in AL. Peaks P1 and P3 were unambiguously identified as neochlorogenic acid and chlorogenic acid via by comparing the retention time and MS/MS fragmentation pattern with those of *mono*-caffeoylquinic acids and the reference standards (Zhang et al., 2015; Willems et al., 2016). The MS/MS spectra of peaks P1 and P3, and possible fragmentation patterns of caffeoylquinic acids are presented in Supplementary Figure S2. These two *mono*-caffeoylquinic acids showed the same deprotonated ion [M-H]^-^ at *m/z* 353.0877 (C_16_H_18_O_9_), and MS/MS ions at *m/z* 191.0560 ([quinic acid-H]^−^), 179.0347 ([caffeic acid-H]^−^) and 135.0452 ([caffeoyl-CO_2_-H]^−^). Similarly, peaks P7 and P8 exhibited the same deprotonated ion [M-H]^-^ at *m/z* 515.1192 (C_25_H_24_O_12_), and MS/MS ions at *m/z* 335.0770 ([M-coffeoyl-H]^-^), 353.0873 ([caffeoylquinic acid-H]^-^), and 299.0558 ([M-coffeoyl-2H_2_O-H]^-^). Comparing the retention time and MS/MS fragmentation pattern with those of *di*-caffeoylquinic acids and the reference standards (Zhang et al., 2015; Willems et al., 2016), peaks P7 and P8 were unequivocally identified as isochlorogenic acid B and isochlorogenic acid A. The MS/MS spectra of these two *di*-caffeoylquinic acids, and possible fragmentation patterns of caffeoylquinic acids are presented in Supplementary Figure S3.

Methoxylated flavonoid is one of the common flavones bearing one or more methoxylated groups on the basic benzo–γ–pyrone (15-carbon, C6–C3–C6) skeleton (Xing et al., 2017). In this present work, five methoxylated flavonoids, including hispidulin (P11), centaureidin (P12), jaceosidin (P13), eupatilin (P14), and casticin (P15) were tentatively identified by comparison of the MS/MS spectras and fragmentation patterns with literatures. Previous studies partially summarized the characteristic fragmentation patterns of polymethoxylated flavones (Ren et al., 2018).

Here, eupatilin (P14) was used as an instance to explain the fragmentation patterns and structural elucidation process of methoxylated flavonoids. The MS/MS spectra and fragmentation patterns of eupatilin are shown in Supplementary Figure S4. In high mass range of the MS/MS spectra, the multiple and alternate loss of •CH_3_ and CO generated several characteristic fragment ions, including *m/z* 328.0587([M-H-•CH_3_]^−^), 313.0350 ([M-H-2×•CH_3_]^−^), 298.0115 ([M-H-3×•CH_3_]^−^), 285.0400 ([M-H-2×•CH_3_-CO]^−^), 270.0164 ([M-H-3×•CH_3_-CO]^−^) and 242.0217 ([M-H-3×•CH_3_-2×CO]^−^). In low mass range of the MS/MS spectra, the fragment ions were generated mainly via RDA cleavage. The fragment ions at 164.0069 and 136.0123 are derived from the RDA cleavage at position 1/3.

In conclusion, the fifteen common peaks in HPLC fingerprints of AL were identified or tentatively identified based on the retention time, MS/MS spectras and fragmentation behaviors by HPLC-Q/TOF-MS. According to the results of qualitative identification, the four potential anti-lipase constitutes in AL represented by peaks P1, P3, P7, and P8 were four caffeoylquinic acids, including neochlorogenic acid, chlorogenic acid, isochlorogenic acid B and isochlorogenic acid A, respectively.

### Verification of Pancreatic Lipase Inhibitory Activity

**FIGURE 6:**
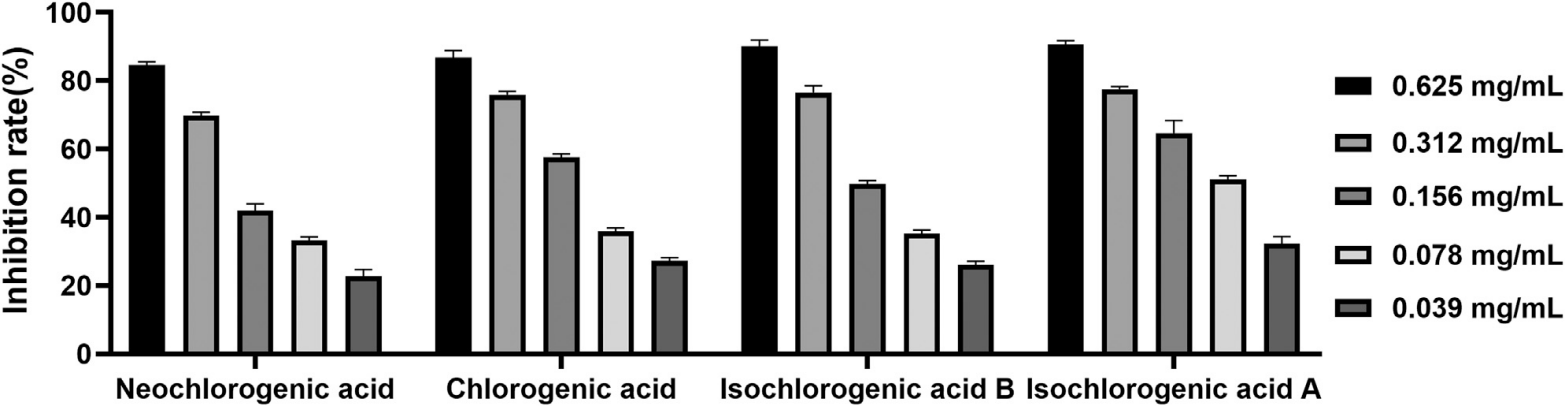
The inhibitory effects of neochlorogenic acid, chlorogenic acid, isochlorogenic acid B and isochlorogenic acid A on pancreatic lipase.

Four caffeoylquinic acids, including neochlorogenic acid (P1), chlorogenic acid (P3), isochlorogenic acid B (P7), and isochlorogenic acid A (P8) were selected as potential pancreatic lipase inhibitors in AL. In order to confirm the reliability of the results, the pancreatic lipase inhibitory capacities of the four constituents were determined by pancreatic lipase inhibition assay. Six concentrations of each compound (0.039, 0.078, 0.156., 0.312, and 0.625 mg/ml) were used. As shown in Figure 6, the four caffeoylquinic acids were found to possess dose-dependent pancreatic lipase inhibitory activities, and the IC_50_ values of neochlorogenic acid, chlorogenic acid, isochlorogenic acid B and isochlorogenic acid A were 0.152 ± 0.020, 0.114 ± 0.025, 0.121 ± 0.022, and 0.081 ± 0.012 mg/ml, respectively. According to the above results, it could be speculated that neochlorogenic acid, chlorogenic acid, isochlorogenic acid B, and isochlorogenic acid A were anti-lipase components of AL.

### Molecular Modeling and Docking Study

**FIGURE 7:**
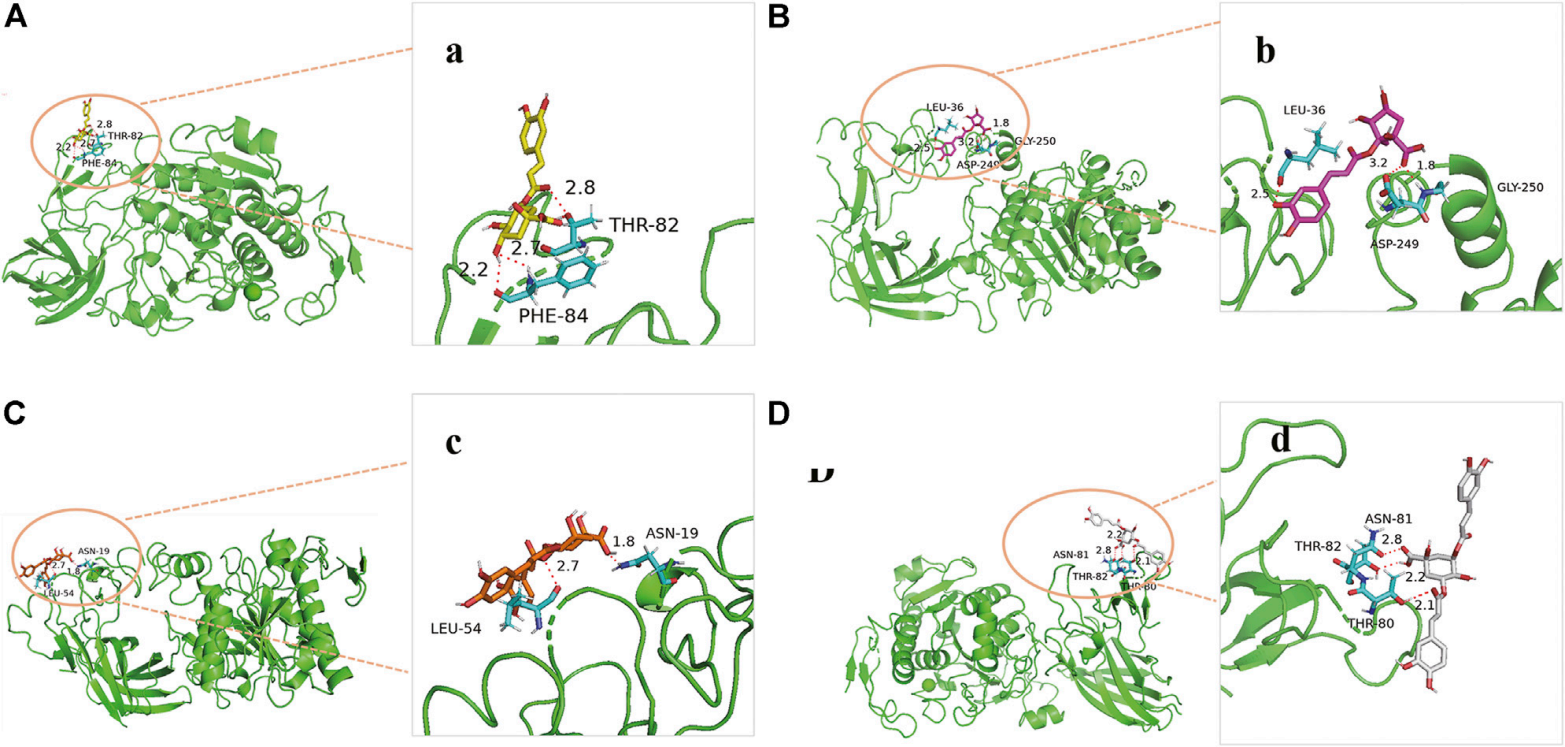
Molecular docking analysis of four anti-lipase compounds with pancreatic lipase. **(A)** Preferred docking position of neochlorogenic acid on pancreatic lipase, **(a)** Interaction of neochlorogenic acid and amino acid residues in pancreatic lipase; **(B)** Preferred docking position of chlorogenic acid on pancreatic lipase, **(b)** Interaction of chlorogenic acid and amino acid residues in pancreatic lipase; **(C)** Preferred docking position of isochlorogenic acid B on pancreatic lipase, **(c)** Interaction of isochlorogenic acid B and amino acid residues in pancreatic lipase; **(D)** Preferred docking position of isochlorogenic acid A on pancreatic lipase, **(d)** Interaction of isochlorogenic acid A and amino acid residues in pancreatic lipase.

In order to predict the preferred binding site between pancreatic lipase and the four anti-lipase compounds, and confirm the results of the pancreatic lipase inhibitory experiments described above, an *in silico* molecular docking study was further performed in this present study (Figure 7). In the process of interaction between neochlorogenic acid and pancreatic lipase, 3-OH on quinic acid groups and carbonyl on caffeic acid groups of neochlorogenic acid could generate hydrogen bonds with Phe84 and Thr82 in pancreatic lipase. Analogously, 3-OH on caffeic acid groups and carbonyl on quinic acid groups of chlorogenic acid could generate four hydrogen bonds with Leu36, His30, Gly250 and Asp249 in pancreatic lipase. Isochlorogenic acid B docked to pancreatic lipase was stabilized by two hydrogen bonds to Asn19 and Leu54, and isochlorogenic acid A docked to pancreatic lipase was stabilized by three hydrogen bonds to Asn81, Thr82, and Thr80. It has been reported previously that the amino acid residues of Ser153, Asp177, and His264 are the key catalytic sites of pancreatic lipase (Huang et al., 2020). However, the results of molecular docking indicated that all the four pancreatic lipase inhibitors in AL did not bind to the three catalytic amino residues, and there were distances from the preferred binding sites of the four inhibitors on pancreatic lipase to the catalytic sites in space.

## Discussion

Hyperlipidemia is a serious public health problem, because hyperlipidemia is closely associated to multiple metabolic diseases, such as diabetes, atherosclerosis and hypertension (Ray et al., 2019). Drug limitation of the intestinal absorption of fats and cholesterol provides a possible approach to prevent or treat hyperlipidemia and other metabolic disorders. Pancreatic lipase is the key enzyme for lipid digestion and absorption, and hydrolysis of triacylglycerols to monoacylglycerols and fatty acids in the duodenum. The pancreatic lipase inhibitors could produce hypolipidemic activity, which could be useful for control or treatment of metabolic disorders (Hou et al., 2020a). Thus, several efforts have been made to discover potential pancreatic lipase inhibiters as anti-lipase agents and a variety of active compounds have been found with inhibitory effects against pancreatic lipase. Among the potential pancreatic lipase inhibiters, orlistat is the most successful case. Orlistat is the only approved pancreatic lipase inhibitor deriving from lipstatin (Nivedita et al., 2018). Although orlistat exhibits good anti-lipase effect, it can lead to several non-negligible gastrointestinal side effects (Pilitsi et al., 2019). The natural phytochemicals existed in some herbal medicines can provide an alternative therapy to reduce the side effects of pharmaceutical drugs (Fabricant and Farnsworth, 2001).

The presence of lipase inhibitors has been reported in several medicinal herbs such as Mori Cortex, Citri Reticulatae Pericarpium Crataegi Fructus and Linderae Radix (Seyedan et al., 2015; Hou et al., 2018; Zeng, et al., 2018). Lots of phytochemicals, especially polyphenols, identified from medicinal herbs exhibited anti-lipase bioactivity (Zheng et al., 2010). As an edible herbal medicine, AL has been widely used in clinic in China and other Asia countries. Our previous studies have shown that polyphenols, including caffeoylquinic acids and flavonoids, are main bioactive constituents in AL, which has been reported to display hypoglycemic and hypolipidemic properties (Guo et al., 2019). However, there is no information about the potential anti-lipase of AL. In this study, the anti-lipase effects of AL were investigated for the first time. The results demonstrated that all the test AL samples exhibited moderate to strong inhibitions on pancreatic lipase with the IC_50_ in the range between 1.92 and 10.29 mg/ml methanol extracts equivalents (Table 2). Taking into consideration that pancreatic lipase is a secreted enzyme in the gastrointestinal tract, the pancreatic lipase inhibitory compounds in AL could directly target on pancreatic lipase after oral administration. It is clear that different batches of AL samples showed significant different anti-lipase activities, which could be owing to the variation of bioactive ingredients in AL samples (Figure 2). Therefore, the pancreatic lipase inhibition results of AL samples encouraged us to further investigate potential anti-lipase compounds from AL.

At present, the multiple chemical components and targets of herbal medicines pose a challenge in discovering bioactive compounds from herbal medicines. The common research methods usually focus on chemical compounds separation and single components activity, which are time-consuming and cannot reveal the complex roles of multiple components in herbal medicines (Quan et al., 2019; Gong et al., 2020). The spectrum-effect relationship is a new and reliable method that can combine chromatographic fingerprint with pharmacological effects by multiple chemometrics. This method could help to explore the correlations between bioactive components and efficacy, and find the major bioactive ingredients in herbal medicines (Yang et al., 2016; Liu et al., 2019). The spectrum-effect relationship method has been successful used to discover varieties of bioactive compounds in herbal medicines. For instance, Wang *et al.* discovered that sinomenine, magnoflorine, menisperine and stepharanine are the major anti-inflammatory compounds in Sinomenii Caulis, and Zeng *et al.* screened out that polymethoxyflavones are the anti-lipase components in Citri Reticulatae Pericarpium by using spectrum-effect relationship method (Zeng et al., 2018; Wang et al., 2019). In this present work, the spectrum-effect relationships between chromatographic fingerprint peaks and anti-lipase activities of AL samples were explored by Pearson correlation analysis and PLSR models. According to the results (Figures 3, 4), the pancreatic lipase inhibition activities were not dominated by one compound but multiple components in AL. The peaks, P3, P8, P1, P7, P2, P10, P6, P9, P4, and P5 showed positive relationships to pancreatic lipase inhibitions, conversely, peaks P11, P12, P13, P14, and P15 had negative relationships. The chemometrics results revealed that the compounds represented by P3, P8, P1, and P7 played important roles in the anti-lipase activity of AL, which could be regard as the pancreatic lipase inhibitors in AL.

The pancreatic lipase inhibitors represented by peaks P3, P8, P1, and P7 were identified as four caffeoylquinic acids (neochlorogenic acid, chlorogenic acid, isochlorogenic acid B and isochlorogenic acid A) by HPLC-Q/TOF-MS. Caffeoylquinic acids, the esters of caffeic acid and quinic acid, are characterized as the important polyphenols present in Asteraceae and Lamiaceae families (Spinola and Castilho, 2017). It has been demonstrated that caffeoylquinic acids could regulate lipid metabolism in genetically and healthy metabolic related disorders (Martinez-Lopez et al., 2019). It is speculated that caffeoylquinic acids can display important roles in regulation of lipid and glucose metabolism and use to treat cardiovascular diseases, diabetes, and obesity (Sotillo and Hadley, 2002; Naveed et al., 2018). Previous investigation has reported that neochlorogenic acid, chlorogenic acid, isochlorogenic acid B and isochlorogenic acid A exhibited dose-dependent inhibitory activities on pancreatic lipase with the IC_50_ values of 1.12 ± 0.04, 1.09 ± 0.08, 0.32 ± 0.02, and 0.39 ± 0.04 mg/ml, respectively (Narita et al., 2012). In our study, the IC_50_ values of the four caffeoylquinic acids were 0.152 ± 0.020, 0.114 ± 0.025, 0.121 ± 0.022, and 0.081 ± 0.012 mg/ml, which are lower than previous research results. The reason for the differences in the results might be due to the different substrates used in the experiments, we used 4-methylumbelliferyl oleate as the water-insoluble substrate, but Narita et al. used triolein. It has been reported that the inhibitory effects of catechins and theaflavins on the pancreatic lipase-catalyzed hydrolysis of triolein were much weaker than those on the pancreatic lipase-catalyzed hydrolysis of 4-methylumbelliferyl oleate (Kobayashi et al., 2009).

To further predict the possible binding sites between pancreatic lipase and the four caffeoylquinic acids, the molecular docking research was performed. It has been reported previously that the amino acid residues of Ser153, Asp177, and His264 are the key catalytic sites of pancreatic lipase (Hou et al., 2020a). However, the results of molecular docking showed that all the four caffeoylquinic acids did not bind to the three key catalytic sites of pancreatic lipase despite their strong anti-lipase activities. It could be speculated that the four caffeoylquinic acids might bind the pancreatic lipase in noncovalent interactions to exert the pancreatic lipase inhibitory effects. In general noncovalent binding between the enzymes and inhibitors is nonspecific and weak, but multiple noncovalent bingings couldchange the conformation and function of the enzymes (Xu et al., 2019). It has been reported that (−)-Epigallocatechin-3-gallate, an anti-lipase component in green tea, which could inhibit the pancreatic lipase without binding the key catalytic sites (Ser153, Asp177, and His264) of pancreatic lipase (Wu et al., 2013).

## Conclusion

In the current study, the HPLC fingerprints of different AL samples were established, and fifteen common peaks were selected. The anti-lipase activities of different AL samples were also evaluated by pancreatic lipase inhibition assay. Then, the spectrum-effect relationships between HPLC fingerprints and pancreatic lipase inhibitory activities of AL samples were firstly investigated to discover the anti-lipase compounds. The Pearson correlation analysis and PLSR results showed a close correlation between fingerprints and anti-lipase activity of AL samples. Four caffeoylquinic acids were selected as potential pancreatic lipase inhibitors in AL, which were identified as neochlorogenic acid, chlorogenic acid, isochlorogenic acid B, and isochlorogenic acid A by HPLC-Q/TOF-MS. Moreover, the pancreatic lipase inhibitory activities of the four caffeoylquinic acids were validated experimentally, and the results indicated that the four bioactive compounds significantly inhibited pancreatic lipase with IC_50_ value ranging from 0.081 to 0.152 mg/ml. The molecular docking study was also performed to predict the preferred binding site between pancreatic lipase and the four anti-lipase compounds in AL. The present study demonstrated the reliability of the spectrum-effect relationships analysis and screened out the anti-lipase compounds in AL. The study provided experimental evidence that AL could be potential herbal medicine and food supplement for treatment of hyperlipidemia or other metabolic diseases. The exact mechanisms of the anti-lipase compounds in AL will be studied in the future.

## Data Availability

The original contributions presented in the study are included in the article/Supplementary Material, further inquiries can be directed to the corresponding authors.
