# Supplementary material for: Screening of Anti-Lipase Components of Artemisia argyi Leaves Based on Spectrum-Effect Relationships and HPLC-MS/MS
Source: Front Pharmacol. 2021 May 7;12:675396. doi: 10.3389/fphar.2021.675396 (PMC8138579; doi:10.3389/fphar.2021.675396)
Supplement: Supplementary file 1 [file DataSheet1.docx]

Supplementary Material


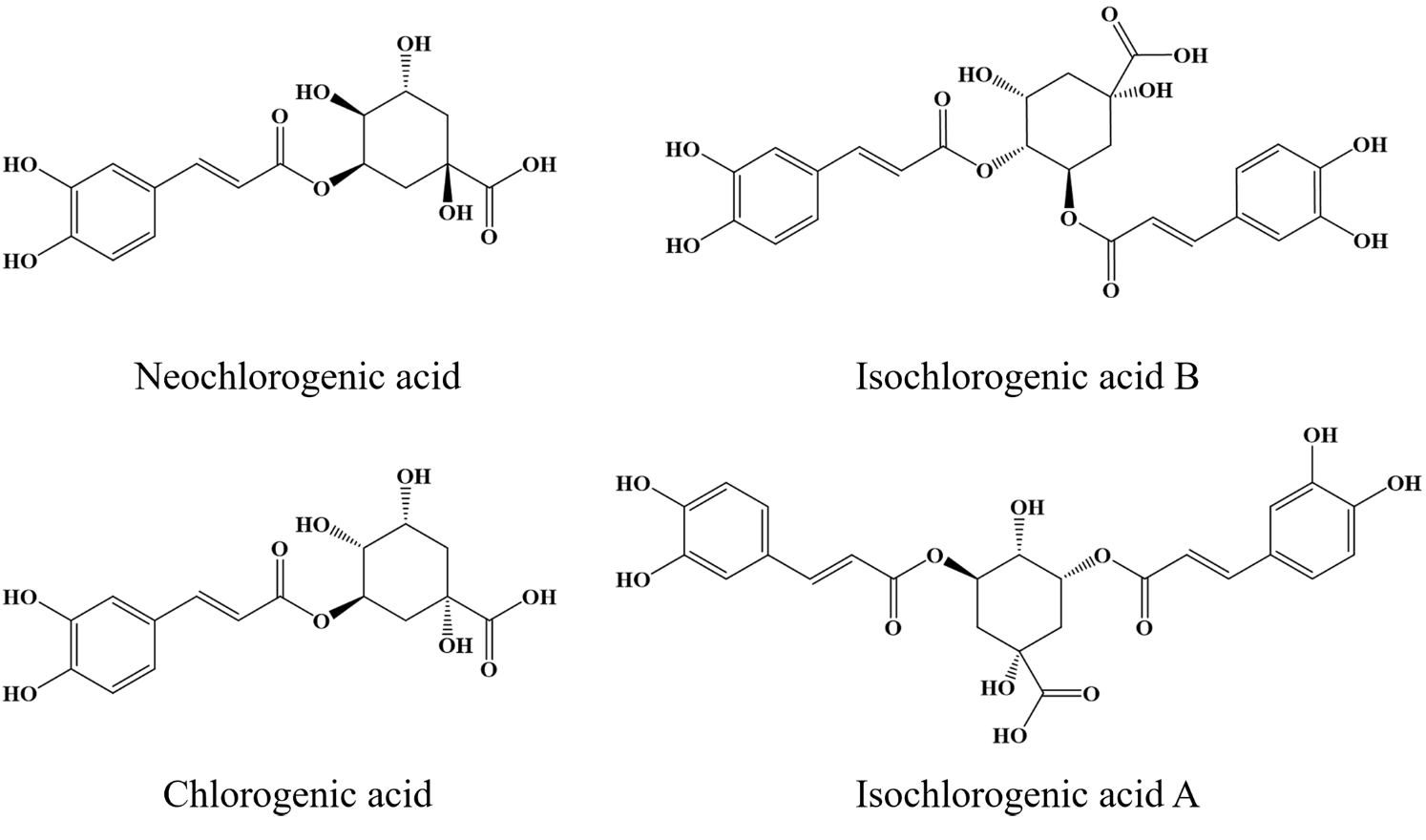


**Supplementary Figure 1** The chemical structures of neochlorogenic acid, chlorogenic acid, isochlorogenic acid B and isochlorogenic acid A.


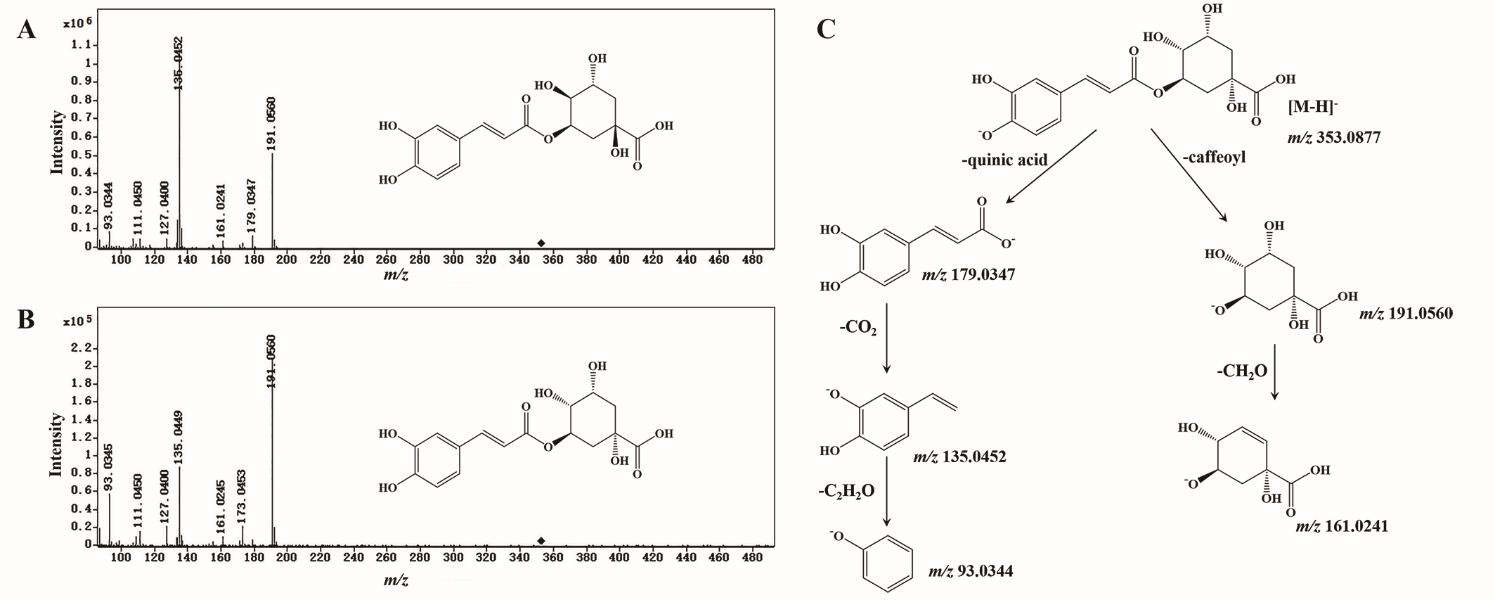


**Supplementary Figure 2** The MS/MS spectra of P1 (A) and P3 (B), and possible fragmentation pattern of *mono*-caffeoylquinic acids (C).


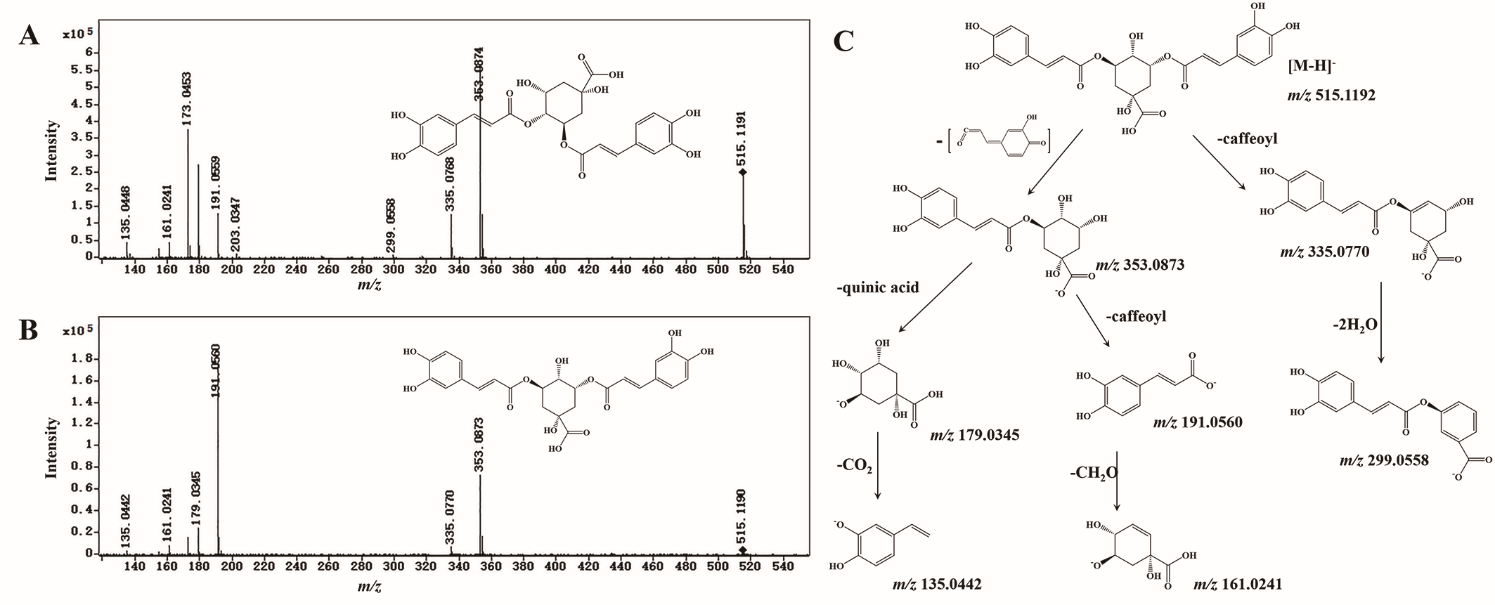


**Supplementary Figure 3** The MS/MS spectra of P7 (A) and P8 (B), and possible fragmentation pattern of *di*-caffeoylquinic acids (C).


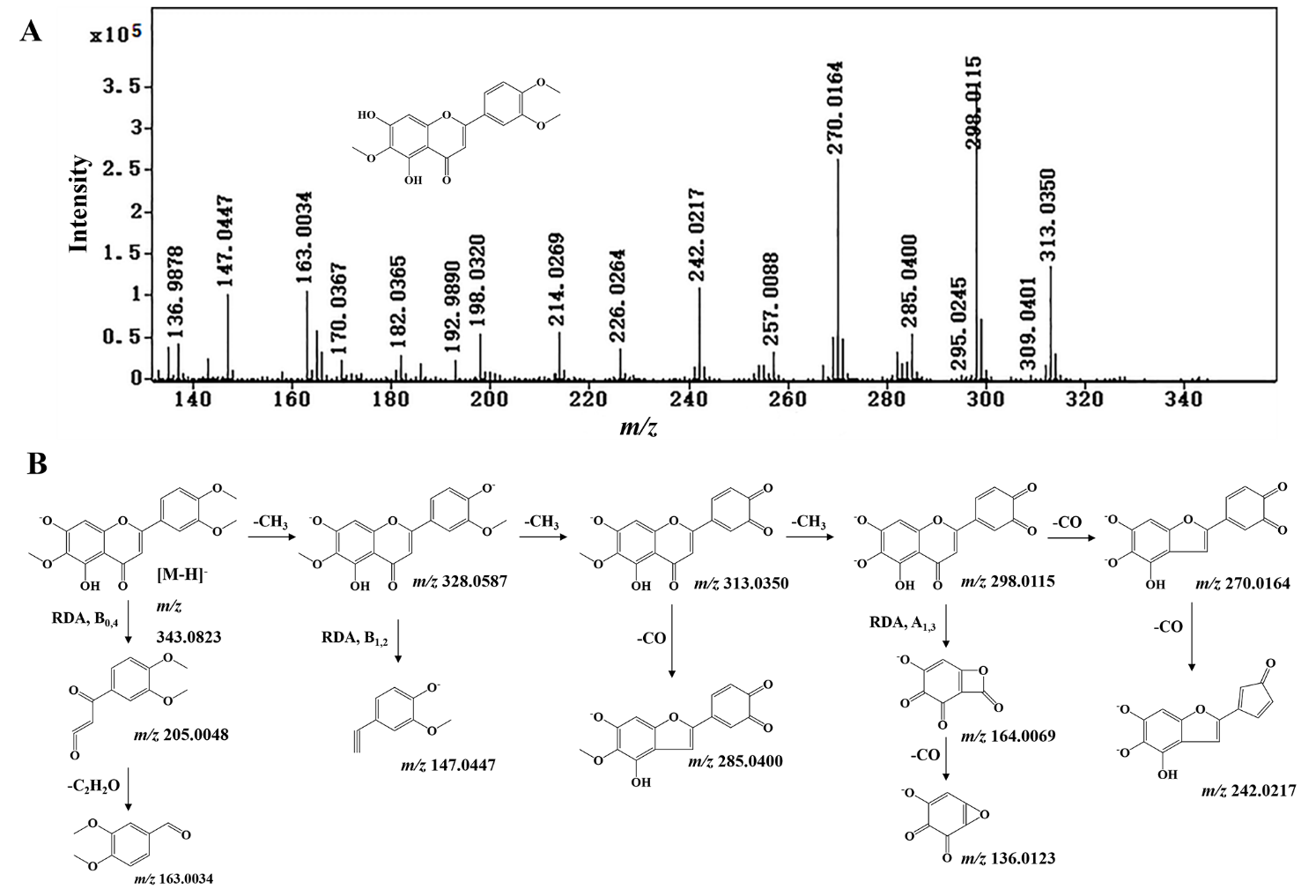


**Supplementary Figure 4** The MS/MS spectrum of P14 (A) and possible fragmentation pattern of methoxylated flavonoids (B).

**Supplementary Table 1** Sample information of twenty-two batches (S1-S22) of *Artemisia argyi* leaves collected from different areas.

| NO. | Origins | NO. | Origins |
| --- | --- | --- | --- |
| S1 | Liuhe Town, Qichun County, Hubei Province | S12 | Guanyao Town, Qichun County, Hubei Province |
| S2 | Cangzhou County, Hebei Province | S13 | Qichun, County, Hubei Province |
| S3 | Xichuan County, Nanyang City, Henan Province | S14 | Neiqiu County, Xingtai City, Hebei Province |
| S4 | Nanyang City, Henan Province | S15 | Xixia County, Nanyang City, Henan Province |
| S5 | Tongbai County, Nanyang City, Henan Province | S16 | Suizhou City, Hubei Province |
| S6 | Laishui County, Baoding City, Hebei Province | S17 | Qingshi Town, Qichun County, Hubei Province |
| S7 | Shizi Town, Qichun County, Hubei Province | S18 | Yi County, Baoding City, Hebei Province |
| S8 | Zhulin Town, Qichun County, Hubei Province | S19 | Yu County, Zhangjiakou City, Hebei Province |
| S9 | Jingxing County, Shijiazhuang City, Hebei Province | S20 | Neixiang County, Nanyang City, Henan Province |
| S10 | Qichun County, Hubei Province | S21 | Xixia County, Nanyang City, Henan Province |
| S11 | Tang County, Baoding City, Hebei Province | S22 | Huangying Town, Nanyang City, Henan Province |

**Supplementary Table 2** The precision, repeatability and stability of the fifteen common peaks (P1-P15) in *Artemisia argyi* leaves.

| Peak | Precision  RSD (%) | | Repeatability  RSD (%)  (n=6) | Stability  RSD (%) |
| --- | --- | --- | --- | --- |
|  | Intra-day (n=6) | Inter-day (n=9)  (n=9) |  |  |
| P1 | 2.2 | 1.4 | 2.3 | 2.7 |
| P2 | 2.3 | 2.3 | 1.5 | 1.2 |
| P3 | 2.2 | 2.8 | 2.6 | 0.8 |
| P4 | 2.5 | 1.0 | 0.7 | 2.2 |
| P5 | 2.1 | 2.1 | 1.5 | 1.5 |
| P6 | 1.5 | 2.2 | 2.3 | 2.2 |
| P7 | 1.8 | 2.6 | 2.5 | 2.8 |
| P8 | 1.7 | 1.4 | 2.1 | 2.4 |
| P9 | 2.0 | 2.9 | 1.5 | 2.6 |
| P10 | 2.5 | 1.3 | 1.3 | 2.1 |
| P11 | 2.3 | 0.5 | 2.6 | 2.0 |
| P12 | 1.8 | 2.2 | 2.4 | 1.8 |
| P13 | 2.4 | 2.7 | 1.8 | 2.3 |
| P14 | 1.3 | 1.8 | 0.9 | 2.4 |
| P15 | 2.7 | 0.9 | 2.6 | 2.0 |
